# Outcomes for a clinically representative cohort of hearing-impaired adults using the Nucleus® CI532 cochlear implant

**DOI:** 10.1007/s00405-020-05893-0

**Published:** 2020-03-05

**Authors:** Matthias Hey, Nicole Neben, Timo Stöver, Uwe Baumann, Alexander Mewes, Tim Liebscher, Mark Schüssler, Antje Aschendorff, Thomas Wesarg, Andreas Büchner, Paula Greenham, Ulrich Hoppe

**Affiliations:** 1 Klinik für Hals-, Nasen-, Ohrenheilkunde, Kopf- und Halschirurgie, Arnold-Heller-Straße 3, Haus 27, 24105 Kiel, Germany; 2 Cochlear Deutschland GmbH & Co. KG, Karl-Wiechert-Allee 76 A, 30625 Hannover, Germany; 3 Klinikum der J. W. Goethe-Universität Frankfurt, Theodor-Stern-Kai 7, 60590 Frankfurt, Germany; 4 Universitätsklinikum Erlangen, Hals-Nasen-Ohren-Klinik, Waldstr. 1, 91054 Erlangen, Germany; 5 Deutsches HörZentrum Hannover der HNO-Klinik der MHH, Karl-Wiechert-Allee 3, 30625 Hannover, Germany; 6 Universitätsklinik für Hals-, Nasen- und Ohrenheilkunde, Killianstrasse 5, 79106 Freiburg, Germany; 7 Greenham Research Consulting Ltd., Downland House, Ashbury, SN6 8LP UK

**Keywords:** Hearing aids, Speech perception, Perimodiolar, Congenital hearing loss, Data logging, Elderly

## Abstract

**Purpose:**

Hearing performance data was collected from a large heterogeneous group of subjects implanted with the Cochlear™ Nucleus® CI532 with Slim Modiolar Electrode, for the purposes of postmarket clinical follow-up. Data was analysed for factors which may predict postoperative speech recognition scores.

**Methods:**

Data was collected retrospectively from five German clinics for 159 subjects from March 2017 to August 2018. Hearing thresholds and recognition scores for monosyllabic words in quiet and sentences in noise were measured preoperatively and at 3 and 6 months postoperatively.

**Results:**

There was a mean gain of 44% points (95% CI 39–49%) at 6 months in monosyllable scores in quiet for implanted ears. Preoperative hearing thresholds in implant ears increased systematically with decreasing age; however, younger subjects had better baseline monosyllable scores with hearing aids compared with older subjects. Baseline performance alone explained 14% of the variation in postoperative scores. Residual hearing was preserved on average to within 22 dB at 250 Hz and 30 dB at 500 Hz of preoperative levels.

**Conclusions:**

In a large and varied cohort of routinely treated hearing-impaired adults, speech recognition with the CI532 for German monosyllabic words in quiet at 6 months was equivalent to performance reported at one year or more in other published studies. Although younger subjects had poorer preoperative pure-tone thresholds, they had better preoperative word recognition scores compared with older subjects, and also had higher post implant scores. Further research is required to identify if this phenomenon is just applicable to German health system assessment and referral practices.

## Introduction

The Cochlear™ Slim Modiolar Electrode (CI532) is a perimodiolar array which was designed to be inserted with minimal damage to the cochlea and to bring the electrode contacts, which deliver the current to the nerve and spiral ganglion cells, closer to the modiolus [1–3].

Improved surgical techniques and cochlear implant (CI) electrode designs minimise cochlear trauma and can allow preservation of residual hearing: When residual hearing is preserved patient outcomes tend to be better, regardless of whether that residual hearing is useable [4–6]. Nonetheless, a recent systematic review by Hoskison et al. [7] shows that trauma to the cochlea does occur in 18% of implantations and in most cases occurs when the electrode array passes from the scala tympani to the scala vestibuli. A growing body of evidence suggests that patients with the electrode array positioned in the scala tympani have the best speech perception outcomes, and outcomes are poorer in patients where the array has dislocated through the basilar membrane and some or all of the electrode contacts reside within the scala vestibuli [2, 4, 8]. Previous studies using the Slim Modiolar electrode array showed consistent perimodiolar placement and scala tympani insertions in at least 87% of the patients assessed with imaging [1–3]. They reported hearing preservation is better than with older perimodiolar and straight designs, but possibly not as good as hybrid or electroacoustic devices that are specifically designed for hearing preservation [2, 3, 9–11].

Positioning the cochlear implant electrode contacts close to the stimulation target reduces the current spread and leads to more focused stimulation; thus, lowering the current levels required to produce behavioural thresholds and comfort levels [12–14]. This localized stimulation provides superior place-pitch spectral discrimination [15, 16], and improved speech perception outcomes [17, 18]. However, due to their increased stiffness and size, perimodiolar arrays which use stylets to keep them straight at the point of insertion, have been associated with poorer hearing preservation results and a higher incidence of basilar membrane trauma compared to thin, flexible lateral wall arrays [19, 20]. The CI532 does not have a stylet and is introduced into the cochlea using a new deployment method called advance-through-the-sheath. It is thinner, less stiff and is 60% of the volume of Cochlear’s previous precurved Contour Advance® array used in the Nucleus CI512; this reduces insertion forces and the potential for trauma [21, 22]. Due its reduced size, it can be inserted through the round window, which has been shown to be beneficial for ensuring initial scala tympani placement and results in better hearing preservation and improved speech perception scores [2, 10, 20]. Previously reported gains in postoperative speech perception and quality of life measures with the Nucleus CI532 are in line with those reported for other devices and are potentially better than with earlier Cochlear devices [3, 10, 23].

It is known that there is a large variation in outcomes for cochlear implant recipients; for example, Lazard et al. [24] identified nine different factors, including age-related effects, such as age at implantation and onset of deafness, as well as duration of severe to profound hearing loss, which influence postoperative speech recognition scores, but their model only explained 22% of the variance [6, 24]. The primary aim of this investigation was to retrospectively collect bench mark hearing performance data in CI532 recipients from five German clinics, reflecting the real-world experience in clinical practice. The aim was to collect data on a cohort of subjects who are representative of the current adult treatment population in Germany. A post hoc exploratory analysis of the potential influence of commonly used patient variables on performance outcomes for speech understanding was performed. We also report hearing preservation for implanted ears with some preoperative residual hearing and compare it to published results for other types of electrode array.

## Method

Data was retrospectively collected for 159 subjects from the clinic databases where at least one speech recognition test was recorded from March 2017 to August 2018. In order to avoid selection bias, records of all subjects implanted with a CI532 device were inspected during the study period and those meeting the inclusion criteria were invited to participate. Subjects could decline to have their data collected without indicating any reasons. The inclusion criteria were: recipients of a CI532 cochlear implant; evaluated with adult hearing tests; good German language skills to assess clinical hearing performance; recipients assessed via routine clinical measurements at pre implant, and at 3 and 6 months postimplant intervals with available data records in their hospital files.

Speech recognition scores for monosyllabic words presented in quiet and sentences presented in noise and unaided hearing thresholds recorded preoperatively and at 3 and 6 months postoperatively were gathered from the patient records, anonymised and entered in the online study database. Bias due to misclassification was addressed by well-defined and monitored data handling procedures. In addition, postoperative imaging indicating electrode placement was summarised by each investigator at each site and entered into a questionnaire. Study subjects followed the routine clinical assessment and management practices in each centre. This included accessing and recording data logging and device characteristics from each sound processor to see how much they had been used in daily life.

Speech recognition in quiet was measured for the implant ear alone (hearing aid before surgery and CI after) using the Freiburg monosyllabic speech test at 65 dB SPL [25] in all centres. However, the materials, test levels and conditions routinely used for speech perception in noise varied across centres. The Hochmair–Schulz–Moser sentence test (HSM) [26] was used with speech at 65 dB SPL and competing noise fixed at a signal to noise ratio (SNR) of +10 dB. Testing in adaptive noise was conducted using the Oldenburg sentence test (OLSA) [27] and Göttingen sentence tests (GÖSA) [28], with speech or noise fixed at 65 dB SPL, depending on the centre.

Standard pure-tone audiometry for frequencies 125–8000 Hz was performed to evaluate residual hearing preimplantation and at 3 and 6 months postimplantation using headphones.

### Compliance with ethical standards

All procedures performed in studies involving human participants were in accordance with the ethical standards of the institutional and national research committee and with the 1964 Helsinki declaration and its later amendments or comparable ethical standards. Ethical Committee approvals were obtained before the start of the study from the ethical committee of each participating clinic. Subjects participating in the study provided formal written informed consent in accordance with the applicable ethical standards for having their pseudonymized data collected before any study-related activities.

### Statistical analysis

Prior to study initiation, a power calculation was carried out to estimate the ability to detect a difference amounting to 20% points, representing a clinically relevant benefit with the Freiburger monosyllabic test, between the preoperative evaluations and 6-month postsurgery evaluation. A sample of *n* = 150 patients was deemed sufficient to detect clinically relevant differences. The primary hypothesis was tested with a one-sample paired *t* test with the difference of the 6-month score minus the preoperative baseline score as outcome. This was applied to percent correct word scores and Speech Reception Threshold (SRT: the SNR in dB at which a 50% correct word score is achieved) values. The results were reported as means with 95% confidence intervals. Due to the differences in clinical practices, not all subjects had a complete set of speech perception measures at each testing interval; hence, a matched group study design was not possible. Missing data points were excluded from this analysis.

An analysis of covariance approach employing linear mixed models was used to elucidate which pretreatment variables could have an influence on 6-month word scores in quiet (primary outcome measure). This allows baseline covariates, such as preoperative score and various biographical variables to be accounted for enabling meaningful average estimates for specific subgroups. The aim was to explore whether the treatment effects hold for the whole study population or are restricted to specific subgroups only. In addition, explanatory variables were checked for covariance. The retrospective nature of the study limits this analysis to the standard information recorded in the clinical notes.

To compute pre- to postoperative differences in hearing thresholds, preoperative values were excluded if they were within 20 dB of the vibrotactile thresholds or audiometer upper limits (i.e. 55 dB HL at 125 Hz, 65 dB HL at 250 Hz, 75 dB HL at 500 Hz, 105 dB HL at 750–8000 Hz) to allow a measurable threshold shift. Patients with preoperative values with no response were removed from the analysis of threshold difference scores and set to “data missing”. Postoperative values greater than the vibrotactile thresholds or audiometer upper limits (i.e. 75 dB HL at 125 Hz, 85 dB HL at 250 Hz, 95 dB HL at 500 Hz) were set to an arbitrary high number, i.e. 999 [29].

Analyses were carried out with the SAS software (SAS Version 9.4, SAS Institute, Cary, NC, USA).

## Results

### Population description

Subject demographics are shown in Tables 1 and 2. Forty-six percent of the sample was female and 54% male. The onset of deafness in the ipsilateral ear was congenital in 12, progressive in 104 and sudden in 36 subjects and not given in 7 subjects. Seven subjects were stated as having a normal hearing ear contralateral to the implant ear and two with asymmetric hearing loss and; therefore, very good contralateral hearing.

**Table 1:** Distribution of age at implantation and duration of hearing loss in implanted ears

	*N*	Mean	Standard deviation	Median	Range
Age at implantation (years)	159	56.6	18.0	58.0	14–91
Duration of hearing loss (years)	148	23.9	18.0	21.0	0–75

**Table 2:** Etiology of hearing loss in the implanted ear

Etiology	Number (percentage)
Chronic otitis media	1 (0.7%)
Cholesteatoma	1 (0.7%)
Familial	4 (2.6%)
Measles	1 (0.7%)
Meniere's disease	4 (2.6%)
Meningitis	2 (1.3%)
Noise	1 (0.7%)
Other^a^	19 (12.5%)
Otosclerosis	5 (3.3%)
Ototoxic drugs	1 (0.7%)
Rubella	2 (1.3%)
Sudden	15 (9.9%)
Unknown	94 (61.8%)
Viral	2 (1.3%)
Missing data	7 (4.4%)
Total	159 (100.0%)

^a^“Other” etiologies included mumps, middle ear surgery, otitis media, Usher syndrome, Waardenburg syndrome, brain hemorrhage in the womb, oxygen deficiency during birth/perinatal, congenital hearing loss and trauma

### Surgical

Surgeons reported that the majority of patients (80%) were implanted using a round window approach. Imaging reports were available for 95% of patients and surgeons indicated that all electrodes in all implants were located in the scala tympani. There were three electrode array tip fold overs reported, which were identified using flat panel CT or digital X-ray. Two cases were corrected at the time of surgery, and one case after postoperative imaging.

### Preoperative hearing status and speech recognition

**Fig. 1:**
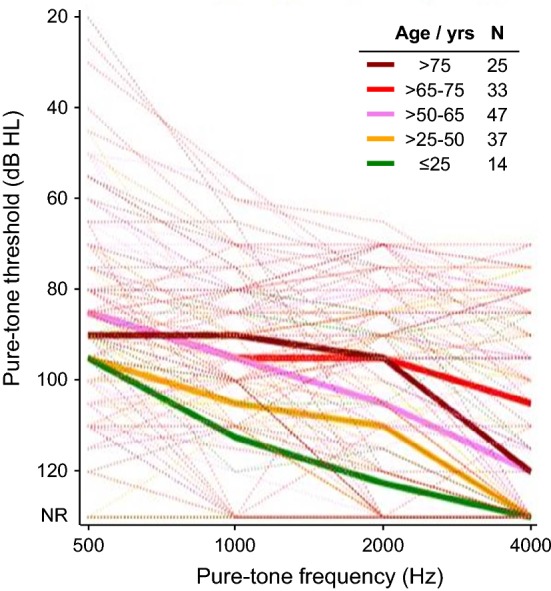
Pure-tone air-conduction thresholds prior to surgery by frequency, for subjects subdivided by age group in years. Median thresholds by group are shown by bold lines. The number of subjects with measurable thresholds at 1000 Hz are given for each group

Implanted subjects presented a range of audiometric thresholds in the ear to be implanted. When thresholds were subdivided by age, there appeared to be a trend towards poorer thresholds with younger age (Fig. 1). Age class was parameterized as linear predictor of threshold with one degree of freedom (DF), and the effect was highly significant (*F* test: *p* < 0.01). The contrast < 50 versus >  = 50 years old proved highly significant yielding a pure-tone threshold difference of ≈ 10 dB HL (SE 3.5) averaged over all frequencies (*t*[152] = 2.8, *p* < 0.01). A principal component analysis of frequencies 500, 1000, 2000, and 4000 Hz identified 1000 Hz as the most dominant pure-tone variable for this analysis. For 1000 Hz alone, the age effect remained significant (*t*[152] = 3.1, *p* < 0.01) with a difference in thresholds of 12 dB HL (SE 4 dB) for those < 50 versus ≥ 50 years.

**Fig. 2:**
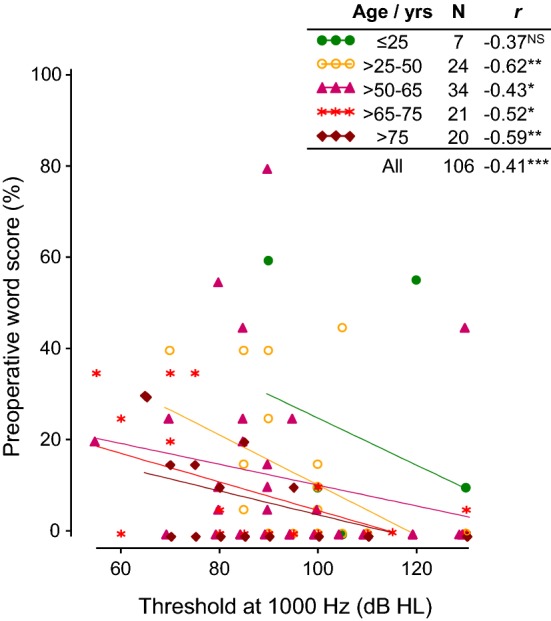
Correlation of air-condition threshold at 1000 Hz with baseline speech perception performance for the ipsilateral ear in quiet for each age group. Spearman’s correlation coefficients (*r*) for each age group in years are given and significant values are indicated with stars; *p < 0.05, **p < 0.01, ***p < 0.001. There is a trend towards better preoperative word scores in the younger subjects

Further analysis showed that, as expected, baseline speech recognition was negatively correlated with threshold at 1 kHz (*r* = − 0.4, *p* < 0.001) for the group as a whole and within-age groups (Fig. 2). Baseline speech recognition scores also indicated a trend towards poorer scores with increasing age at testing even though thresholds were better with increasing age at testing. There appeared to be a covariance between baseline speech perception, age and threshold with younger subjects having better speech recognition with their hearing aids despite having poorer preoperative thresholds relative to the older subjects. The pattern of data was not amenable to reliable further parametric linear modelling of preoperative scores with age group and threshold as factors.

### Postoperative speech recognition in quiet

One hundred and forty-seven subjects had recorded speech recognition scores for monosyllables in quiet for the implant ear at the 6-month interval (Table 3): Mean scores at 6 months for the group were 55% correct (50–75%). There was a significant difference between baseline preoperative scores and 6-month scores for the implanted ear of 44.2% points (39.3–49.1%) and between preoperative scores and 3-month scores of 37.3% points (32.1–42.6%.) There was also a significant difference between 3- and 6-month scores of 7.2% points (4.3–10.0%).

**Table 3:** Distribution of percent correct word scores for speech perception with ipsilateral ear in quiet by visit and change in scores between preoperative values and 3- and 6-month monosyllable scores (within patient) for ipsilateral ear in quiet

Visit	Number	Mean	SD	Median	Low CL	Up CL	Min	Max	*t*	*p* value
Pre	109	9.8	16.3	0.0	6.7	12.9	0.0	80.0		
3 M	144	48.1	27.5	45.0	43.6	52.6	0.0	100.0		
6 M	147	54.9	24.8	55.0	50.9	59.0	0.0	100.0		
Diff: 3 M—pre	96	37.3	26.1	37.5	32.1	42.6	− 35.0	100.0	14.0	< 0.0001
Diff: 6 M—pre	99	44.2	24.5	45.0	39.3	49.1	− 10.0	90.0	17.9	< 0.0001
Diff: 6 M—3 M	135	7.2	16.8	5.0	4.3	10.0	− 32.5	55.0	4.97	< 0.0001

*CL* confidence limit, *SD* standard deviation

**Fig. 3:**
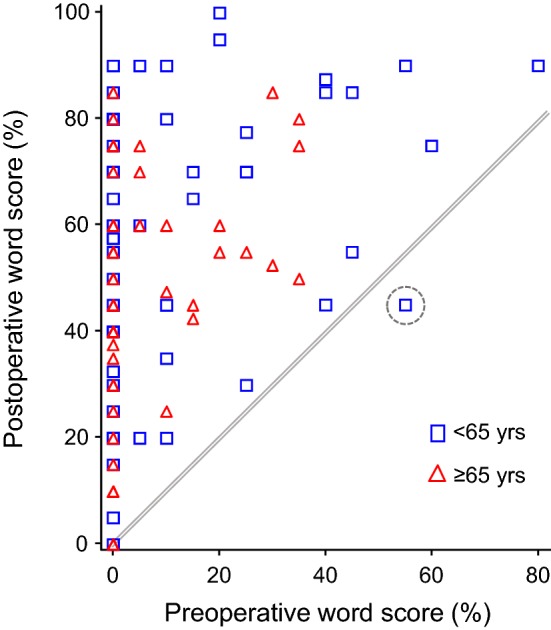
The relationship between preoperative and postoperative word scores for the implanted ear. Postoperative scores are at 6 months, or at 3 months where 6-month data was not available. All scores above the line represent equivalent or better postoperative compared to preoperative performance

Figure 3 shows that the large majority of subjects (96%) had higher postoperative word scores compared to preoperative scores in the implanted ear. We note that 46 subjects had zero percent correct word scores preoperatively, but then a large range of postoperative outcomes (range 0–90% correct).

**Fig. 4:**
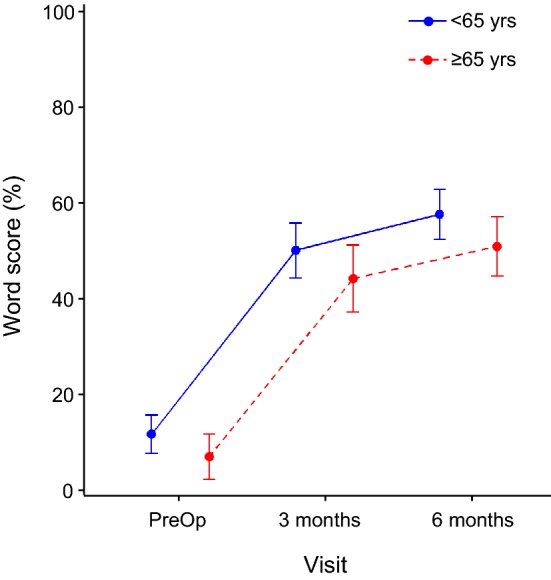
Word scores for the ipsilateral ear in quiet for each visit divided into age groups of less than and more than or equal to 65 years of age. Points show the least squares means and whiskers the 95% confidence intervals of the mean

Postoperative speech recognition scores were initially grouped by younger than 65 and 65 years and older because in most developed countries, the medical term ‘elderly’ is roughly defined as retirement age, that is, over 65 years old. The sample consisted of 95 subjects less than 65 years and 64 greater than or equal to 65 years. Over all visits, patients ≥ 65 years had lower mean word scores as compared to patients < 65 years, the difference amounting to 5.2% points. A linear mixed model using all data, with subject as random variable and visit and age group as fixed effects, revealed a trend for age group (*t*[136] = 1.87, *p* = 0.064). Removing the subjects with congenital hearing loss produced a statistically significant age effect (*t*[128] = 2.51, *p* = 0.013). The younger subjects maintained better word scores, as seen in the baseline data, across all visits (Fig. 4). There was no statistically significant interaction effect between age and visit, which is clear from Fig. 4. Thus, while the degree of improvement over time is similar for all ages, younger subjects begin with higher speech scores preimplant and maintain these higher scores at both postimplant test intervals, relative to the older group. As noted above, nine subjects had single-sided deafness or asymmetric hearing impairment (four in the younger group, five in the older group): their scores were in line with those obtained across all subjects, with slightly higher mean score for the younger group at 3-month postimplant (50% versus 45% correct).

### Regression analysis of speech recognition scores in quiet

An explorative prediction analysis was carried out for speech recognition scores in quiet in the ipsilateral ear condition at 6 months postsurgery as the outcome measure. Potential predictive variables included were: gender, duration hearing loss, age at implantation, age at onset of initial hearing loss, previous ear operations, tinnitus in the past 12 months, dizziness/vertigo, aetiology of hearing loss and onset of hearing loss. We also included pure-tone thresholds, as in the above analysis of baseline performance. The included variables were limited by the retrospective nature of the study.

Duration of hearing loss and age at onset of hearing loss were the only factors that emerged as significant. However, these two variables were highly correlated showing an inverse relationship (*r* = − 0.63, *p* < 0.001) so two models were constructed. Duration of hearing loss explained marginally more variation (*R*^2^ = 0.0629) so was used going forward. If, however, baseline performance was also included in the model, the effect of duration of hearing loss was no longer statistically significant. Baseline performance alone explained 14% of the variation in postoperative scores (*R*^2^ = 0.1358, *n* = 91, *p* < 0.001). When preoperative scores of zero were disregarded, the relationship remained (*r* = 0.31, *p* < 0.05, *n* = 40). This significant correlation reflects the fact that subjects performed as well or better postoperatively compared with preoperatively.

The impact of the small group of 10 subjects with congenital hearing loss, who had postoperative speech scores, warranted further investigation. Ten congenital subjects were in the 25–50 age group and had the lowest age at onset of hearing loss (4.8 years compared to a mean of 32.3 years). They did not do significantly worse than the rest of the group (median speech perception scores 57.5% for postlingual group and 37.5% for congenital group, Wilcoxon two sample test *p* = 0.18) and onset of hearing loss did not emerge as a significant factor in the regression analysis for 6-month scores in quiet.

However, if improvement from before surgery to 6 months is considered in a stepwise logistic regression model, using imputed preoperative values set to 0 for missing baseline values, both duration of hearing loss and congenital hearing loss were significant. The calculated odds ratio showed a roughly sevenfold lower chance of improvement for subjects with congenital hearing loss compared to patients with no congenital hearing loss. With regard to duration of hearing loss, the odds of improvement were lowered by a factor of 2 per 10 years.

### Speech recognition in noise

Speech in noise tests are not routinely performed where word scores in quiet are already very low. In this sample, only 18 subjects had preoperative speech recognition scores in noise for the ipsilateral ear. Of these eight were in fixed noise and twelve adaptive. This meant that assessing the improvement of speech recognition in noise over time was severely compromised by missing data and not considered to be a valid analysis. These missing score in noise have been caused by very low preoperative speech recognition scores in quiet and therefore obsolete measures in noise not to further frustrate the CI candidates while assuming even worse scores. Thus, mean values for speech in noise at 6 months postoperative only are reported.

At 6 months, mean speech perception for the ipsilateral ear in +10 dB SNR noise was 24% correct (15–32%) based on a subgroup of 36 subjects who had been tested. In the adaptive tests, mean scores for the ipsilateral ear are shown in Table 4. Speech recognition thresholds in noise obtained with the OLSA were significantly lower than for the GÖSA (Wilcoxon test *p* = 0.044). This is likely reflective of test difficulty (type of sentence and noise) and not group performance per se.

**Table 4:** Signal to noise ratio at which 50% correct speech perception was achieved (speech reception threshold—SRT) for the GÖSA and OLSA speech tests for the implanted ear

Test condition	Number	Mean	SD	Median	25th percentile	75th percentile	Low CL	Up CL	Min	Max
GÖSA	28	6.1	9.4	4.9	− 0.7	13.6	2.4	9.7	− 7.1	33.7
OLSA	29	1.3	3.9	0.4	− 0.6	2.7	− 0.2	2.8	− 4.3	15.3

*SD* standard deviation, *CL* confidence limit

### Preservation of residual hearing

**Fig. 5:**
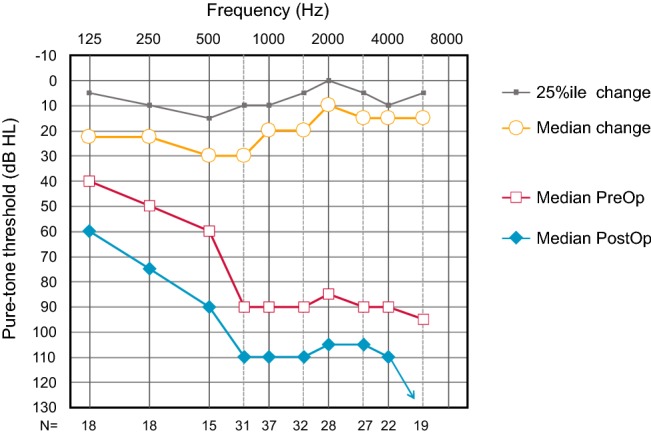
Median preoperative and 6-month postoperative pure-tone thresholds. At the bottom of the figure, the numbers are given for cases with matched data at each frequency for all subjects with measurable thresholds preoperatively

Figure 5 provides the data for subjects with preoperative thresholds in the ipsilateral ear and 6-month threshold at each frequency. Due to the number of “no response” values entered as 999 the median threshold at 8000 Hz at 6 months could not be calculated. Table 5 shows the number of subjects who had measurable thresholds at 6 months postsurgery compared to the number who had measurable thresholds before surgery.

**Table 5:** Number of subjects who had measurable thresholds by frequency presurgery with at least 20 dB headroom and who still had measurable thresholds at 6 months postsurgery, i.e. not vibrotactile or exceeding the limits of the audiometer

Frequency Hz	125	250	500	750	1000	2000	4000
Pre-op	18	18	15	31	37	28	22
6-months	11	11	10	18	27	23	13

The change in thresholds from pre- to post-surgery reached statistical significance for each frequency (Wilcoxon signed rank test, *p* < 0.01 with Bonferroni correction for multiple comparisons). However, changes from 3 to 6 months did not reach significance.

### Processor use and acoustic environment

Sound processor data loggings were downloaded for 146 subjects at 6 months and showed how long each day subjects used their device. There was no difference in time-on-air for older versus younger subjects, or by gender: At the 6-month evaluation subjects < 65 years had used their processors for an average of 12.3 h a day (SD 3.4, *n* = 88) and those ≥ 65 years for 11.3 h per day (SD 3.4, *n* = 58). Overall, it appears that the proportions of the times spent on air at the various sound levels were different between patients < 65 years and ≥ 65 years. This was true for the 3-month and 6-month visits. A multivariate test over all levels by age class indicated a significant difference (Wilks’ Lambda, *p* < 0.0001). Proportions over time were significantly different across all sound levels except > 50 < 60 dBA. The highest differences were found for sound levels > 60 and < 70 dBA, and ≥ 70 and < 80 dBA. That is, older subjects spent less time in these sound levels compared to younger subjects and less time per day in noisy situations (as classified by the processor).

## Discussion

There was a clinically and statistically significant improvement of 44% points in speech perception in quiet between preoperative and 6-month scores for the implanted ear for the group. There was also a small, but significant improvement between 3 and 6-month postoperative scores of seven percentage points for the group. In order to put the results into context, mean scores for the group at 6 months were compared to the results reported in the literature for the same German speech perception measures. Studies were included from the last 10 years investigating CI patients with modern coding strategies in use in the last 2 decades and comparable to today’s CI devices. Mean group results were reported for the Freiburg monosyllable test in quiet at 1 year in a single centre by Lenarz et al. [30–32] and at 6 months by Hast et al. [33] and at a mean use of 3 years by Haumann et al. [34] and ranged from 45 to 63% correct. Study cohorts had mean ages of 50–60 years and included older patients over 75 years, so comparable to this study sample with a mean age of 57 years. The 55% correct score reported in our multicentre study falls well within the range and represents results from a real-world treatment cohort across multiple centres, including patients with congenital and acquired loss and single-sided deafness. Single centre prospective studies allow for greater control over confounding variables, but the results are not always representative of what can be expected from a broader clinical sample in real-world practice. The results are encouraging at the 6-month point, and further improvement in some subjects can be expected over the course of the first year of implant use and beyond [5, 35]. The results in noise were compromised by the high-missing data rates and the number of different tests used. This resulted in small sample sizes for each of the noise tests used and reduced the applicability of the results to a wider population.

It is also possible to consider the hearing preservation results in the context of published data with both the CI532 and other perimodiolar electrodes. However, this is more complicated than for speech perception as there is great variation in the way results are reported with fewer standards for defining the parameters which contribute to hearing threshold calculation. Factors to be taken into consideration are the frequencies included, the way in which missing data or thresholds which exceed the limits of the audiometer are handled and whether it is possible to aid any preserved hearing. In this study missing values or values exceeding audiometer limits were entered as 999, as recommended by Fraysse et al. [29], increasing the mean and median values for the group. Subjects were not selected as hearing preservation candidates, but some did have good low frequency hearing before surgery and the low frequency average of 125, 250 and 500 Hz for the analysis sub-group was ~ 50 dB HL. In comparison to other hearing preservation data reported for the CI532, median losses of less than 30 dB HL for a low frequency average of 250 and 500 Hz were larger than those reported in the literature [9–11, 36, 37]. Mean low frequency shifts were considerably better compared to the older CI512 device [4, 29] and in line with values reported for lateral wall and mid-scala electrode arrays [10, 19, 38, 39]. This is in agreement with the recent data reported by Holder et al. [10] showing slightly better hearing preservation for the CI532 compared to a matched group of users of the Cochlear CI422 and 522 straight electrode arrays.

A large variability in the speech recognition results was observed, even though the data set represented a group of subjects with the same electrode array positioned within the scala tympani. As all clinics tested patients in quiet using the Freiburger monosyllables, a multi-regression analysis was conducted on this outcome measure to look for factors which might contribute to this variability. The regression analysis revealed that speech recognition scores at 6 months will be higher for better baseline scores, and that this factor was the strongest predictor for this sample. Preoperative performance alone accounted for 14% of the outcome variation at 6 months in this sample. Duration of hearing loss a significant factor when considered as a sole variable, but was no longer significant once baseline speech performance was added into the model. Holden et al. [17] and Hoppe et al. [6] also identified preoperative speech score as a predictor of postoperative performance, but other studies have not shown this relationship [2, 4]. However, Lazard et al. [24] showed that the preoperative score was not an independent variable, but was influenced by the age at onset, etiology, hearing aid use and pure-tone audiogram. Clinically, however, baseline performance alone is not useful as an indicator of postoperative speech recognition, especially for subjects who have zero speech recognition scores before surgery [6]. This is evidenced in this sample by the subjects with zero preoperative scores, some of who went on to be high scorers on postoperative speech tests. Hearing threshold in the ipsilateral ear was also good predictor of preoperative speech perception (*r* = − 0.41), but a poorer predictor of performance at 6 months post implant (*r* = − 0.24). This is in line with Carlson et al. [4] who also showed low frequency pure tone average was similarly weakly correlated to postoperative performance (*r* = 0.2) and Lazard et al. [24] who found that better thresholds in the implanted ear were related to better postoperative outcomes.

If we look at the improvement in speech perception made after surgery, which is the most clinically relevant factor for potential CI candidates, only duration of hearing loss and having an acquired loss rather than a congenital loss are weak predictors for this. Younger subjects performed better with monosyllabic words at baseline and maintained this advantage at the 3- and 6-month testing intervals over the older age group as expected. Other authors have noted a relationship between a younger age and better postoperative performance [4, 18, 40–42]. Holden et al. [18] even found that once scalar location had been removed as a variable, age was the only factor that still correlated significantly with outcomes. This trend towards better speech recognition in younger patients is not unique to cochlear implant users. The monosyllabic word scores of older hearing aid users are significantly lower than for younger adults [43] and the same is true for speech perception in noise [44]. This had been assumed to be as a consequence of poor quality amplification, but even where amplified speech was presented via headphones there was still a reduction in word recognition ability with increasing age [43]. When thresholds were investigated further in our cohort, younger patients experienced worse preoperative thresholds compared to older patients across the frequency range, which would tend to reduce their preoperative speech scores with hearing aids (such as seen here in Fig. 2). However, pure-tone thresholds alone are just one of many factors affecting speech comprehension, and cognitive decline that naturally occurs with age has an impact. Older subjects have to cope with the additional burden that presbycusis places on central auditory processing ability, with additional dysfunction either in the auditory cortex or in signal processing within the brainstem, which may decrease performance on speech recognition tests [45].

Duration of processor use was the same for both the younger and older groups. This is a good indicator of self-perceived benefit as patients are unlikely to continue using a device if they do not feel it is beneficial. Data logging also showed that there was a small but statistically significant difference between the length of time younger subjects spent in louder and noisier environments, with older subjects tending not to be exposed to noisier situations. It would be interesting to explore if this difference exists due to avoidance of noise by older subjects or differences in the exposure to noise due to lifestyle factors.

### Limitations

It was not possible to exclude potential bias from self-selection (subjects who refused to participate) or loss to follow-up (subjects with incomplete records). Not all subjects had speech recognition measures at each testing interval and missing data points were excluded from the analysis. This introduces a bias towards better performing subjects as those poorer performing patients are the ones which are most likely not to be tested to reduce patient stress, especially in noise. The retrospective nature of the study limited the factors which could be considered in the analysis of variance. For example, hearing aid use before implantation and educational level, both of which are known predictors of better postoperative performance, were not recorded in the study data base and were therefore not considered [17, 24]. The method for analyzing the audiometric threshold shift can be considered as very conservative and may have led to a larger threshold shift compared with other analysis methods.

## Conclusions

Speech recognition with the CI532 for German monosyllabic words in quiet at 6 months was equivalent to performance reported at 1 year or more in other published studies for a large and varied cohort of routinely treated hearing-impaired adults.

Cochlear implantation with the Nucleus CI532 results in equivalent benefit in word recognition scores for older and younger subjects. Older subjects also used their processor for the same amount of time as younger subjects, supporting the finding of equivalent benefit, but tended not to be exposed to louder and noisier environments. Predictors for postoperative word scores were age at testing and word scores in the preimplant listening condition. Younger subjects had better preoperative speech recognition performance on word tests and higher post implant speech scores at 3 and 6 months than older subjects. However, younger subjects had worse preoperative pure-tone thresholds than the older subjects. Further research is required to identify if this phenomenon is just applicable to German health system assessment and referral practices, or if it is a wider issue across cultures, potentially related to the better capacity of younger subjects to use any remaining hearing for preoperative speech recognition.
